# Pharmacological suppression of Nedd4-2 rescues the reduction of Kv11.1 channels in pathological cardiac hypertrophy

**DOI:** 10.3389/fphar.2022.942769

**Published:** 2022-08-17

**Authors:** Hua Zhang, Tian Fu, Jinglei Sun, Sihao Zou, Suhua Qiu, Jiali Zhang, Shi Su, Chenxia Shi, De-Pei Li, Yanfang Xu

**Affiliations:** ^1^ Department of Pharmacology, The Key Laboratory of New Drug Pharmacology and Toxicology, Hebei Medical University, Shijiazhuang, Hebei Province, China; ^2^ Center for Precision Medicine, Department of Medicine, School of Medicine University of Missouri, Columbia, MO, United States

**Keywords:** rapid delayed rectifier K+ current, pathological cardiac hypertrophy, Nedd4-2, ubiquitination, arrhythmia

## Abstract

The human *ether-á-go-go-related* gene (hERG) encodes the pore-forming subunit (Kv11.1), conducting a rapidly delayed rectifier K^+^ current (*I*_Kr_). Reduction of *I*_Kr_ in pathological cardiac hypertrophy (pCH) contributes to increased susceptibility to arrhythmias. However, practical approaches to prevent *I*_Kr_ deficiency are lacking. Our study investigated the involvement of ubiquitin ligase Nedd4-2-dependent ubiquitination in *I*_Kr_ reduction and sought an intervening approach in pCH. Angiotensin II (Ang II) induced a pCH phenotype in guinea pig, accompanied by increased incidences of sudden death and higher susceptibility to arrhythmias. Patch-clamp recordings revealed a significant *I*_Kr_ reduction in pCH cardiomyocytes. Kv11.1 protein expression was decreased whereas its mRNA level did not change. In addition, Nedd4-2 protein expression was increased in pCH, accompanied by an enhanced Nedd4-2 and Kv11.1 binding detected by immunoprecipitation analysis. Cardiac-specific overexpression of inactive form of Nedd4-2 shortened the prolonged QT interval, reversed *I*_Kr_ reduction, and decreased susceptibility to arrhythmias. A synthesized peptide containing the PY motif in Kv11.1 C-terminus binding to Nedd4-2 and a cell-penetrating sequence antagonized Nedd4-2-dependent degradation of the channel and increased the surface abundance and function of hERG channel in HEK cells. In addition, *in vivo* administration of the PY peptide shortened QT interval and action potential duration, and enhanced *I*_Kr_ in pCH. We conclude that Nedd4-2-dependent ubiquitination is critically involved in *I*_Kr_ deficiency in pCH. Pharmacological suppression of Nedd4-2 represents a novel approach for antiarrhythmic therapy in pCH.

## Introduction

Pathological cardiac hypertrophy (pCH) is an enlargement of the myocardium in response to various pathological stimuli including hypertension, ischemia, and cardiomyopathy. The pCH may eventually lead to ventricular dilation and heart failure. Cardiac electrical remodeling in pCH or heart failure predisposes the heart to ventricular arrhythmias and increases the risk of sudden cardiac death (Tomaselli and Zipes, 2004). Thus, the mechanisms underlying electrical remodeling in hypertrophied and failing hearts have been studied extensively (Tomaselli and Marban, 1999; Cutler et al., 2011). Ion channel dysfunction is a hallmark of electrical remodeling in pCH and heart failure. However, traditional antiarrhythmic medications directly targeting ion channels are likely to impose proarrhythmic side effects on hypertrophied or failing hearts (Echt et al., 1991). Therefore, developing precision antiarrhythmic therapeutics to rescue cardiac ion channel function is an urgent need.

Delayed ventricular repolarization is one of the distinct electrophysiological changes in either hypertrophic or failing hearts and is reflected by prolonged action potential duration (APD) and QT interval (Marban, 2002; Li et al., 2004; Hegyi et al., 2018). The delayed repolarization is prone to trigger early afterdepolarizations (EAD) and leads to exaggerated spatial and temporal heterogeneities of ventricular repolarization, both of which render the heart more susceptible to deleteriousness tachyarrhythmia (Wallace et al., 2019). Accumulated experimental data suggest that dysfunction of K^+^ channels in pCH is a major cause of cardiac electrical remodeling and arrhythmias (Marban, 2002; Li et al., 2004; Asbun and Villarreal, 2006; Hegyi et al., 2018). The delayed rectifier K^+^ currents (*I*_K_), consisting of rapidly and slowly activating components (*I*_Kr_ and *I*_Ks_), respectively, are the major repolarizing currents in ventricular action potentials in humans and other large mammals. The human *ether-a-go-go*-related gene (*hERG*) encodes K^+^ channel pore-forming subunit (Kv11.1) conducting *I*_Kr_ current is crucial for the repolarization in cardiac action potentials (Shenasa and Shenasa, 2017). A reduction of *I*_Kr_/hERG currents due to either genetic defects or adverse drug effects can lead to hereditary or acquired long-QT syndrome in humans characterized by an increased risk for ventricular arrhythmias and sudden cardiac death (Marban, 2002). It has been shown that a decrease in *I*_Kr_ is involved in APD prolongation and contributes to arrhythmogenesis in pCH or heart failure (Furukawa and Kurokawa, 2006; Rahm et al., 2018). However, the molecular mechanism underlying *I*_Kr_ deficiency remains largely unknown.

Ubiquitination, the first step for membrane protein internalization, requires activating E3 ubiquitin ligase Nedd4-2 (Goel et al., 2015). Nedd4-2 binds to a conserved PY motif of the target proteins and delivers ubiquitin to the lysine residues to initiate their internalization and degradation through proteasome or lysosome pathway (Lamothe and Zhang, 2016). It has been identified that hERG possesses a typical PY motif in its C-terminal region (Cui and Zhang, 2013; Lamothe and Zhang, 2016). Overexpression of Nedd4-2 in heterologous HEK293 cells downregulates hERG expression in the plasma membrane and thus decreases hERG currents *via* enhancing hERG ubiquitination (Guo et al., 2012). On the other hand, inhibition of Nedd4-2 activity through phosphorylation induced by overexpression of serum and glucocorticoid inducible kinase SGK1 or SGK3 increases cloned hERG expression and currents and the native *I*_Kr_ (Lamothe and Zhang, 2013). It has been shown that Nedd4-2 expression levels are significantly increased in the myocardium of rats with heart failure induced by volume overload and in hypertrophied neonatal rat cardiomyocytes (Luo et al., 2017). Increased Nedd4-2 level promotes the degradation of Nav1.5 channel and reduces sodium current (*I*_Na_) densities since Nav1.5 channel also contains a PY motif in its C-terminal tail (van Bemmelen et al., 2004; Luo et al., 2017). Therefore, we proposed that increased Nedd4-2 activity in pCH enhanced ubiquitylation of Kv11.1 and accelerated its degradation, leading to *I*_Kr_ reduction and consequent arrhythmogenesis. We used multi-scale approaches to test this hypothesis in angiotensin II (Ang II)-induced guinea pig pCH model. Based on our findings, we developed a novel pharmacological approach to prevent the downregulation of *I*_Kr_ in pCH through effectively suppressing Nedd4-2-dependent ubiquitination.

## Materials and methods

### Animal models of pathological cardiac hypertrophy and *in vivo* interventions

Male adult guinea pigs weighing 250–300 g were purchased from Vital River Laboratory Animal Technology Co., Ltd. (Beijing, China). Animals were acclimatized to their new environment for 1 week before the experimental procedure. Under anesthesia with isoflurane (2%, RWD Life Science Co., China), guinea pigs were implanted with osmotic pumps (Alzet-2002, ALZA Corporation, United States) in the back of the neck containing either saline (CON) or angiotensin II (Ang II, MCE, United States) at a dose of 0.6 mg/kg/d for 14 days. Animals were housed at 25°C, 50%–60% relative humidity, with a 12 h light/dark cycle. All animals were fed a standard laboratory diet and filtered tap water *ad libitum*. All animal care and experimental procedures were approved by the Animal Care and Ethical Committee of Hebei Medical University (Shijiazhuang, China). Some animals were intravenously administrated with deno-associated virus vectors containing the Nedd4-2 mutant at 4 weeks before Ang II treatment. In another group of guinea pigs, the synthetic PY peptide was intravenously treated (0.23 mg/kg every 2 days for 14 days) at the beginning of Ang II adminstration.

### Echocardiography and *in vivo* electrocardiographic recordings

Guinea pigs were anesthetized with 1–2% isoflurane, and transthoracic two-dimensional (2D) guided M-mode echocardiography was performed using an 18-38 MHz probe (Vevo^®^ 2,100 Imaging System, FUJIFIUM VisualSonics Inc., Toronto, Canada). The thickness of the anterior and posterior walls of the left ventricle and interventricular septum during the systolic and diastolic periods were measured. Left ventricular ejection fraction (LVEF) and left ventricular fractional shortening (LVFS) were calculated. For ECG recordings, the lead II waveforms were recorded in guinea pigs under anesthetized with 1–2% isoflurane using the Biopac 150 System (Biopac Systems, United States). The ECG parameters were measured and averaged over 20 consecutive beats per acquisition to determine heart rate (defined by RR intervals) and the QT interval (defined as the time between the beginning of the QRS complex to the end of the T wave). The corrected QT intervals (QTc) for the heart rate were calculated according to Bozzat equation QTc = QT×(333/RR)^0.601^

### Langendorff isolated heart perfusion and programmed electrical stimulation

The Langendorff hearts were prepared using the same method as described before (Lv et al., 2015). Retrograde perfusion was performed (5–6 ml/min; 37 ± 0.5°C) through the aorta with oxygenated Tyrode’s solution using a peristaltic pump. The hearts were placed in a thermostatic chamber and allowed to equilibrate for a minimum of 30 min to ensure stable ECG recordings before electrical stimulation. The *in vitro* equivalent to lead II ECG waveforms were recorded using the Biopac 150 System (Biopac Systems, Goleta, CA, United States) at 5 kHz. The stimulating electrode (Biopac Systems, United States of America) was connected to impose a series of currents stimulation in 10 Hz and lasted 2 ms. The stimulation started from 50 mA and increased by 10 mA for every step until arrhythmia occurred. The ventricular tachycardia threshold (VTT) was measured, and the incidence of arrhythmia under a specific current stimulation was observed (Wang et al., 2014).

### Isolation of ventricular cardiomyocytes

Single ventricular myocytes were enzymatically dissociated from the hearts of guinea pigs as described previously (Lv et al., 2015). In brief, guinea pigs were anesthetized with isoflurane (2%) after intraperitoneal injection of heparin (1,000 units/kg). The hearts were extracted quickly and mounted on a Langendorff apparatus to be perfused with Ca^2+^-free Tyrode’s solution (in mM) containing NaCl 140, KCl 5.4, MgCl_2_ 1, HEPES 10, and glucose 10 in mM, pH adjusted to 7.4 with NaOH, and followed by Ca^2+^-free Tyrode’s solution containing type II collagenase (0.5 mg/ml, Worthington Biochemical Co., Lakewood, NJ). The hearts were removed from the perfusion apparatus once ventricular tissue softened and the free wall of the left ventricular was cut into small pieces, which were placed in high K^+^ solution (in mM) containing KOH 80.0, KCl 40.0, KH_2_PO_4_ 25.0, MgSO_4_ 3.0, Glutamic acid 50.0, Taurine 20.0, HEPES 10.0, EGTA 0.5, Glucose 10.0 in mM, pH adjusted to 7.3 with KOH. Single cardiomyocytes were then harvested and used for patch-clamp recording within 4–6 h after isolation.

### Patch-clamp recordings

The Whole-cell patch-clamp technique was used to record action potentials and K^+^ currents from dissociated guinea pig ventricular myocytes. Action potentials were evoked at a rate of 1 Hz with suprathreshold current pulse of 2.0 nA for 4 ms duration applied *via* patch electrodes in the current-clamp mode. The APD was measured at 50% and 90% repolarization (APD_50_ and APD_90_). The pipette solution contained (in mM) KCl 140, Mg-ATP 4, MgCl_2_ 1, EGTA 5, and HEPES 10 (pH 7.2 with KOH). The external solution contained (in mM) NaCl 138, KCl 4, MgCl_2_ 1, CaCl_2_ 2, NaH_2_PO_4_ 0.33, glucose 10 and HEPES 10 (pH 7.4 with NaOH). The conventional V-clamp mode was used to record *I*_*Kr*_ and *I*_*Ks*_ currents. Borosilicate glass electrodes (VitalSense Scientific Instruments Co., Ltd. China) had tip resistances of 1–3 MΩ when filled with the pipette solution containing (in mM) KCl 140, Mg-ATP 4, MgCl_2_ 1, EGTA 5, and HEPES 10 (pH 7.2 with KOH). The external solution contained (in mM) NaCl 132, KCl 4, CaCl_2_ 1.8, MgCl_2_ 1.2, glucose 5 and HEPES 10 (pH 7.4 with NaOH). Na^+^ and T-type Ca^2+^ currents were inactivated by a holding potential of −40 mV. Nimodipine (Nim, 1 μM, Sigma, United States) was added to the external solution to block the L-type Ca^2+^ current. E4031 (2 μM, Cayman Chemical, United States) was added into the external solution to block *I*_Kr_ during *I*_Ks_ recording, and HMR-1556 (2 μM, Tocris, United Kingdom) was used to block *I*_Ks_ when *I*_Kr_ was recorded (Thomas et al., 2003). All experiments were performed at room temperature (24–25°C) using an Axopatch 700B amplifier (Molecular Devices, United States). The electrical signals were recorded at sampling frequency of 2.5–10 kHz, filtered at 1-2 kHz using a low-pass filter and digitized with an A/D converter (Digidata 1322; Molecular Devices, United States). Membrane capacitance was measured, and the tail current amplitudes were normalized to cell membrane capacitance to obtain tail current densities (pA/pF). The pClamp software (Version 10.2; Molecular Devices) was used to generate protocols and acquire and analyze the data.

### Quantitative PCR

Total RNA was extracted from tissues of the free wall of left ventricular using Trizol Reagent (Takara, Aomori-Pref, Japan). The quantity of RNA was determined spectrophotometrically (NanoDrop 1000, Thermo Scientific, Wilmington, DE, United States), and the purity of RNA was confirmed by relative absorbance at 260 *versus* 280 nm. cDNA was synthesized according to the PrimeScript™ RT Reagent Kit (Takara, Japan) instructions. Real-time PCR was performed using a fluorescence temperature cycler (ABI 7500 real-time PCR instrument, ABI, Wilmington, DE, United States). The following primers (Guinea pig-derived) (synthesized by Sangon Biotech, Shanghai, China) were used: GAPDH forward: 5’-AAG​ACC​TTG​GGC​TGG​GAC​T-3’; GAPDH reverse: 5’-CCA​AAT​CCG​TTG​ACT​CCG​AC-3’. ANP forward: 5’-CTG​TGA​CGG​GCT​GAG​GTT​GT-3’; ANP reverse: 5’-TGG​CAA​GTT​TGT​GCT​GGA​AG-3’. β-MHC forward: 5’-ATG​CTG​GCA​CCG​TGG​ACT-3’; β-MHC reverse: 5’-TTA​GGA​GCT​TGA​GGG​AGG​ACT​T-3’. TGF-β forward: 5’-AAG​AAG​TCA​CCC​GCG​TGC​TA-3’; TGF-β reverse: 5’-TGT​GTG​ATG​TCT​TTG​GTT​TTG​TCA-3’. ERG forward: 5’-TGC​TAC​AGA​GGC​AGA​TGA​CG-3’; ERG reverse: 5’-GAA​AGC​GAG​TCC​AAG​GTG​AG-3’. KCNQ1 forward: 5’-TCA​GGC​GCA​TGC​AGT​ACT​TT-3’; KCNQ1 reverse: 5’-GAT​TCG​CAC​CAT​GAG​GTT​GA-3’.

The amplification curves to provide Ct values were normalized to the reference gene GAPDH; the changes in expression were calculated using the 2−ΔΔCT method.

### Immunoblotting and immunoprecipitation

The left ventricular tissue was lysed in RIPA buffer (Solarbio life sciences technology Co., Ltd. China) containing inhibitors for protease and phosphatase (or ubiquitination inhibitor, MG132, MCE, United States) on ice for 20 min. The tissue lysis was centrifuged at 12,000 g for 30 min at 4°C; then, the pellet was discarded. Protein concentration was measured by the BCA protein assay kit (Thermofisher, United States). Denatured samples (40–60 μg/lane) were separated on 10% SDS-PAGE and transferred onto a PVDF membrane (Roche, Germany). Membranes were blocked with 3% BSA for 1 h and incubated with primary antibodies overnight at 4°C. After being washed, membranes were incubated with goat anti-rabbit (611-145-002) or anti-mouse (610-145-121) secondary antibodies (1:10000, Rockland Immunochemicals, United States). Quantification of the signals was performed by Odyssey Infrared Imaging System (LICOR 9120, Li-COR, United States). The protein bands were normalized to the GAPDH bands in each sample. Antibodies against Kv7.1 (APC-022), KCNE1 (APC-163), or Kv11.1 (APC-016) were obtained from Alomone Labs, Jerusalem, Israel. The anti-Nedd4-2 (13690-1-AP), anti-Rab11 (15903-1-AP), and anti-GAPDH (10494-1-AP) antibodies were purchased from Proteintech, China. The antibodies against ubiquitin (ab7780) and p-Nedd4-2 (ab168349) were obtained from Abcam, United Kingdom. For the co-immunoprecipitation (Co-IP) experiment, the protein samples from left ventricular tissues containing 1.0 mg protein were incubated with 10 μg IgG (Santa Cruz sc-2027, United States of America) or designated antibodies overnight at 4°C, and then incubated with protein A/G PLUS-Agarose beads (Santa Cruz, United States) for 6 h at 4°C. After being washed with RIPA buffer, immunoprecipitated proteins were eluted from agarose beads by 5 × SDS sample buffer (Life Technology, United States) and denatured at 95°C for 10 min. Immunoprecipitated proteins were run in SDS-PAGE, immunoblotted, and detected with corresponding antibodies.

### Generation and administration of recombinant adeno-associated virus vectors

Recombinant AAV (rAAV) vector (serotype 9, AAV-9) containing the Nedd4-2 (C801S) mutant gene and carrying enhanced green fluorescent protein (eGFP) sequences driven by the cardiac troponin T (cTnT) promoter were constructed by Weizhen Biotechnology Co., Ltd. China. The viral vectors at a titer of 8 × 10^11^ viral particles were injected intravenously into guinea pigs at a volume of 100 μl. The expression of eGFP was confirmed under a microscope equipped with fluorescent illuminance after 4 weeks and then Ang II was administered as described above.

### Cell culture and treatment

Human embryonic kidney (HEK) cells with stable expression of hERG gene (hERG-HEK 293 cells) were cultured in Dulbecco’s modified Eagle’s medium (DMEM, Gibco) supplemented with 10% fetal bovine serum (Gibco) and G418 (400 μg/ml) with 5% CO_2_ at 37°C. The hERG-HEK 293 cells were first transiently transfected with different quality of Nedd4-2 (NM_001144964) plasmid (500, 1000, or 2000 ng) using Lipofectamine 2000 (Invitrogen) according to the manufacturer’s instructions, and then cultured for 48 h after completely replacing the culture medium. Our self-designed PY peptide sequence is -mtlvppaysavt-, and the randomly generated scrambled peptide sequence is -vmpaltyvtasp-. The PY peptide (synthesized by Beijing Zexiyuan Technology Co., Ltd. China) was fused with cell-penetrating peptide (--Arg-Lys-Lys-Arg-Arg-Gln-Arg-Arg-Arg) and TRITC fluorophore. The hERG-HEK 293 cells were collected and subjected to western blot analysis or patch-clamp recordings after administration of PY peptides in the culture medium at final concentrations of 100, 200, and 500 nM for 48 h.

### Flow cytometry

As previously described (Aromolaran et al., 2014), we constructed a tagged wild-type (WT)-hERG plasmid, in which a 13-residue high-affinity bungarotoxin binding site (BBS) tag was introduced into the extracellular S1-S2 loop of the channel to label the surface hERG channels with fluorescence CY5-conjugated bungarotoxin (BTX-CY5). YFP was fused to the C-terminus of hERG channels to enable simultaneous fluorescence detection of total hERG expression. Human embryonic kidney (HEK293) cells transiently transfected with WT-BBS-hERG-YFP displayed robust fluorescence signals for total (YFP) and surface (BTX-CY5) hERG protein expression. After the WT- BBS-hERG-YFP was transfected (2 μg/well) for 24 h, Nedd4-2 (1,000 ng) and PY peptide (500 nM) were applied. Cells were incubated with Brefeldin A (BFA, 10 μM, MCE, NJ, United States) at different time points (0, 4, 12 and 20 h), and harvested after being gently washed by PBS. Then, α- Bungarotoxin-CY5 (5.0 μg/ml, Bosun life, China) was used to stain cells at a temperature of 4°C in a dark place for 1 h. BD LSRII (BD Biosciences, United States) Cell Analyzer was used to analyze data. We utilized untransfected and single-color controls to set the appropriate gain for each fluoro-phore to ensure that signals remained linear in order to set threshold values. The same gain setting was used for assaying each group of experiments.

### Cardiac histology

Heart was fixed in 4% paraformaldehyde followed by dehydration and embedding in paraffin wax, then was sectioned into slices at a thickness of 5–8 microns. These sections were mounted on glass slides and dried on a 45°C hot plate for 30 min. The tissues were subjected to hematoxylin and eosin (H&E) staining to assess the hypertrophic cardiomyocyte area, and Masson staining to assess the degree of myocardial fibrosis. The cell cross-sectional area and fibrosis area were measured and quantified by CaseViewer software (3DHISTECH, Hungary).

### Statistical analysis

All statistical analyses were performed using GraphPad Prism 7 (GraphPad Software, Inc.) and SPSS statistical software version 20 (SPSS, Inc.). The data were presented as mean ± SD. Comparisons between groups were performed using unpaired Student’s *t*-test or one-way ANOVA with Dunnett’s and Tukey’s post-hoc test. A Chi-square test was used to compare the incidence between groups. Mann–Whitney test or Kruskal-Wallis test was used to compare data with skewed distribution. The results with *p* < 0.05 were considered statistically significant.

## Results

### Nedd4-2-dependent ubiquitination was involved in the downregulation of *I*_Kr_ in pCH

Consistent with the previous studies (Wang et al., 2014), we found that administration of Ang II into guinea pigs for 2 weeks induced a typical pCH and electrical remodeling. The wall thickness of anterior, posterior, and ventricular septum along with myocyte sizes and myocardial fibrosis area were significantly increased (Supplementary Figures S1A,B); the weight ratios of the whole heart/body weight and left ventricle/body weight were significantly higher but the weight ratio of lung/body weight remained unchanged (Supplementary Figure S1C); myocardial mRNA levels of hypertrophic biomarkers including *ANP*, *β-MHC,* and *TGF-β* were significantly increased (Supplementary Figure S1D). However, the ejection fraction (EF) and short-axis shortening rate (FS) did not differ from vehicle-treated animals (Supplementary Table S1), indicating that cardiac function was reserved and not progressed to the heart failure stage. The QT intervals and the heart rate corrected QTc were prolonged in pCH guinea pigs (Supplementary Figures S1E,F). And isolated ventricular cardiomyocytes of pCH guinea pigs displayed a remarkably longer APD (both APD_50_ and APD_90_ were significantly prolonged, Supplementary Figures S1G,H). In addition, 11 of 29 pCH guinea pigs died within 2 weeks altogether without showing any signs of disease (sudden death), while all vehicle-treated guinea pigs were survived (Supplementary Figure S1I). To determine the susceptibility of cardiac arrhythmia, the ventricular tachycardia threshold (VTT) was determined by applying the programmed electrical stimulation to isolated Langendorff-perfused hearts (Supplementary Figure S1J). The VTT values in pCH guinea pigs were significantly lower than those in the control group (Supplementary Figure S1L). In addition, a 130-mA stimulation did not trigger arrhythmias in hearts from control guinea pigs, whereas it induced arrhythmias in 5 of 6 pCH guinea pigs (Supplementary Figure S1K).

**FIGURE 1:**
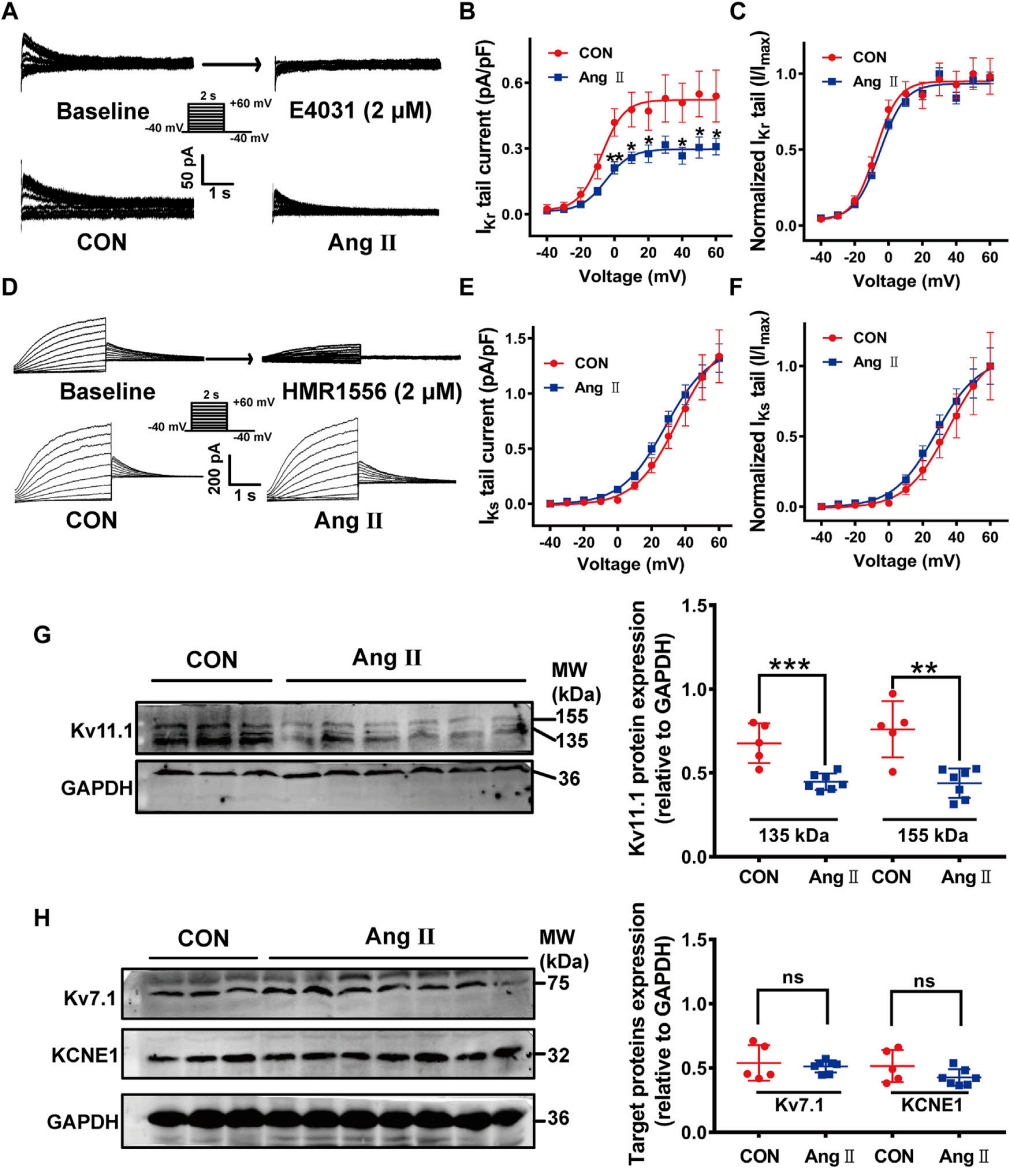
*I*_Kr_ currents and Kv11.1 protein were reduced in the myocardium in pCH. **(A)** Representative tail traces of *I*_Kr_ before (left) and after application of E-4031 (2 μM) (right) in upper panel. Ventricular myocyte was pulsed as shown protocol. The lower panel showing representative tail traces recorded in myocytes from control (CON) and pCH (Ang II), respectively. **(B)** Summary data for *I*_Kr_ tail current density-voltage relationship in control and pCH (*n* = 10–14 cardiomyocytes from 3 to 5 hearts, *p < 0.05 *versus* CON). **(C)** Normalized current-voltage relationship for *I*_Kr_ tail current. Curves were fit by Boltzmann function. **(D)** Representative *I*_Ks_ traces recorded using the pulse protocol shown in the inset before (left) and after application of HMR1556 (2 μM) (right) in upper panel. The lower panel showing representative traces recorded in myocytes from control (CON) and pCH (Ang II), respectively. **(E)** Summary data for *I*_Ks_ tail current density-voltage relationship in control and pCH (*n* = 11–15 cardiomyocytes from 3 to 5 hearts). **(F)** Normalized current-voltage relationship for *I*_Ks_ tail current. Curves were fit by Boltzmann function. **(G)** Representative immunoblots bands for mature and immature Kv11.1 proteins and corresponding summary data (CON *n* = 5, Ang II *n* = 7). **(H)** Representative immunoblots bands for Kv7.1 and KCNE1 proteins and corresponding summary data (CON *n* = 5, Ang II *n* = 7). GADPH was used as an internal control to normalize these bands. *p < 0.05, **p < 0.01, and ***p < 0.001 *versus* CON. ns: not statistically significant.

We next recorded *I*_Kr_ and *I*_Ks_, which are critically determine APD in isolated left ventricular myocytes of guinea pig hearts. As shown in Figure 1A, *I*_Kr_ was recorded from ventricular myocytes, which were depolarized from a holding potential of −40 mV to a serial of pre-pulse potentials from −40 to +60 mV in increments of 10 mV for 2 s and repolarized to −40 mV to evoke outward tail currents in the presence of the *I*_Ks_ blocker HMR1556 (2 µM). The tail currents were abolished by a specific *I*_Kr_ blocker E-4031 (2 µM) (Figure 1A upper panel). Much smaller *I*_Kr_ tail currents were observed (Figure 1A lower panel) and current densities at most of the depolarization potentials were significantly decreased in pCH animals compared to control (*p*< 0.05 at voltages from +10 mV to +60 mV, Figure 1B). However, there was no difference for the voltage-dependent activation of the channel between two groups (Figure 1C). Meanwhile, *I*_Ks_ was recorded in the presence of *I*_Kr_ blocker E4031 by using the same voltage protocol for *I*_Kr_ recording and was verified by the *I*_Ks_ specific blocker HMR1556 (Figure 1D upper panel). Representative *I*_Ks_ current traces showed no obvious difference between pCH and control animals (Figure 1D lower panel). There was no significant difference in *I*_Ks_ density at recorded potentials and voltage-dependent activation of the channel between control and pCH animals (Figures 1E,F). Observations suggested that decreased *I*_Kr_, but not *I*_Ks_ contributed to the APD prolongation in pCH. Then we determined the expression levels of Kv11.1 and Kv7.1 proteins, which carry *I*_Kr_ and *I*_Ks_ in the myocytes, respectively. Immunoblots for Kv11.1 proteins extracted from the left ventricular myocardium displayed two bands at 155 and 135 kDa, representing the mature fully glycosylated form in the plasma membrane (155 kDa) and the immature core-glycosylated form residing in the endoplasmic reticular (135 kDa), respectively (Lamothe et al., 2016) (Figure 1G). Both mature and immature Kv11.1 protein levels were significantly lower in pCH myocardial tissue (Figure 1G). Proteins expression for the *I*_Ks_ channel subunits KCNQ1 (Kv7.1) and KCNE1 were not changed (Figure 1H). On the other hand, the mRNA expression level of *KCNH2* gene, which encodes Kv11.1, did not significantly differ between pCH and control guinea pigs (Supplementary Figure S2), whereas *KCNQ1* gene was significantly increased in pCH myocardium (Supplementary Figure S2). These results suggested that a posttranscriptional mechanism is involved in the downregulation of Kv11.1/*I*_Kr_ in pCH hearts.

**FIGURE 2:**
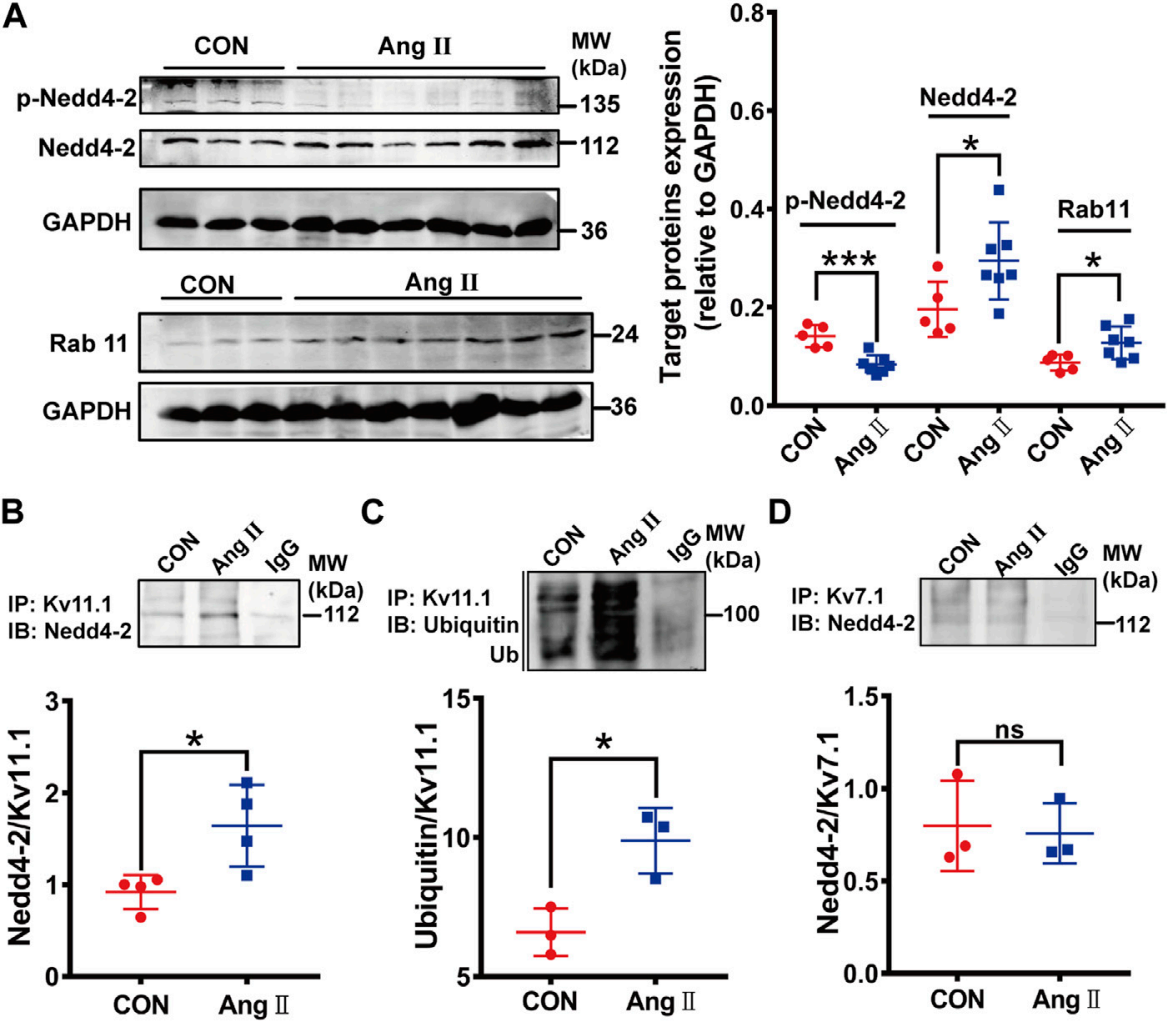
Nedd4-2-mediated ubiquitination of Kv11.1 was increased in pCH. **(A)** Representative immunoblots showing Nedd4-2, phosphorylated Nedd4-2 (p-Nedd4-2) and Rab11 proteins and corresponding quantifications of band densities in myocardium from control (CON) (*n* = 5) and pCH (Ang II, *n* = 7). GADPH was used as an internal control to normalize the bands. **(B)** Representative blots immunoprecipitated (IP) by anti-Kv11.1, and western immunoblotting (IB) was performed by using anti-Nedd4-2 antibody. Quantification of band densities was shown as the ratio of Nedd4-2 to Kv11.1. **(C)** Representative blots immunoprecipitated by anti-Kv11.1, and western immunoblotting (IB) was performed by using anti-ubiquitin antibody. Quantification of band densities was shown as the ratio of ubiquitin to Kv11.1. **(D)** Proteins from control and pCH myocardium were immunoprecipitated with an anti-Kv7.1 or anti-IgG antibody, and IB was performed by using anti-Nedd4-2 antibody. IgG was used as negative controls. *n* = 3–4. *p < 0.05 and ***p < 0.001 *versus* CON. ns: not statistically significant.

We determined if Nedd4-2-dependent ubiquitination was involved in the downregulation of Kv11.1/*I*_Kr_ through measuring expressions of Nedd4-2 and phosphorylated Nedd4-2 (p-Nedd4-2), the inactivated form (Lamothe and Zhang, 2016). Western blotting analysis showed that the Nedd4-2 protein level was significantly higher, while p-Nedd4-2 was lower in pCH myocardium than those in control guinea pigs (Figure 2A). These data suggest that Nedd4-2 activity is increased in pCH. We further determined if Nedd4-2 protein directly interacts with Kv11.1 channel through co-immunoprecipitation. Immunoblotting bands using antibodies against Nedd4-2 or ubiquitin were significantly increased in protein complexes immunoprecipitated (pulled down) by an antibody against Kv11.1 channel in pCH hearts compared with control (Figures 2B,C). This finding supported the notion that the interaction between Nedd4-2 and Kv11.1 was enhanced in pCH. In addition to Kv11.1, Kv7.1 is another substrate of Nedd4-2 since Kv7.1 also contains Nedd4-2 binding PY motif (Jespersen et al., 2007; Lamothe and Zhang, 2016). Thus, we detected the interaction between Nedd4-2 and Kv7.1. Intriguingly, although the Kv7.1 antibody pulled down Nedd4-2 protein, the immunoprecipitated protein complex using antibody against Nedd4-2 did not significantly alter in pCH compared with the control group (Figure 2D).

In addition to Nedd4-2, a small GTPase, Rab11 promotes internalized Kv11.1 channels to recycle to the cell membrane (Delisle et al., 2009; Lamothe and Zhang, 2013). Thus, disruption of endogenous Rab11 by overexpressing the Rab11 dominant-negative mutants significantly decreases the 155-kDa hERG expression level and hERG current (Chen et al., 2015). We then measured the Rab11 expression and found that Rab11 expression level was higher in pCH myocardium than in the control group (Figure 2A), suggesting that Rab11 is unlikely involved in reducing *I*_Kr_. The high expression of Rab11 may be a secondary response to an increment of internalized hERG channels.

### Effects of suppressing Nedd4-2 on electrical remodeling in pCH

**FIGURE 3:**
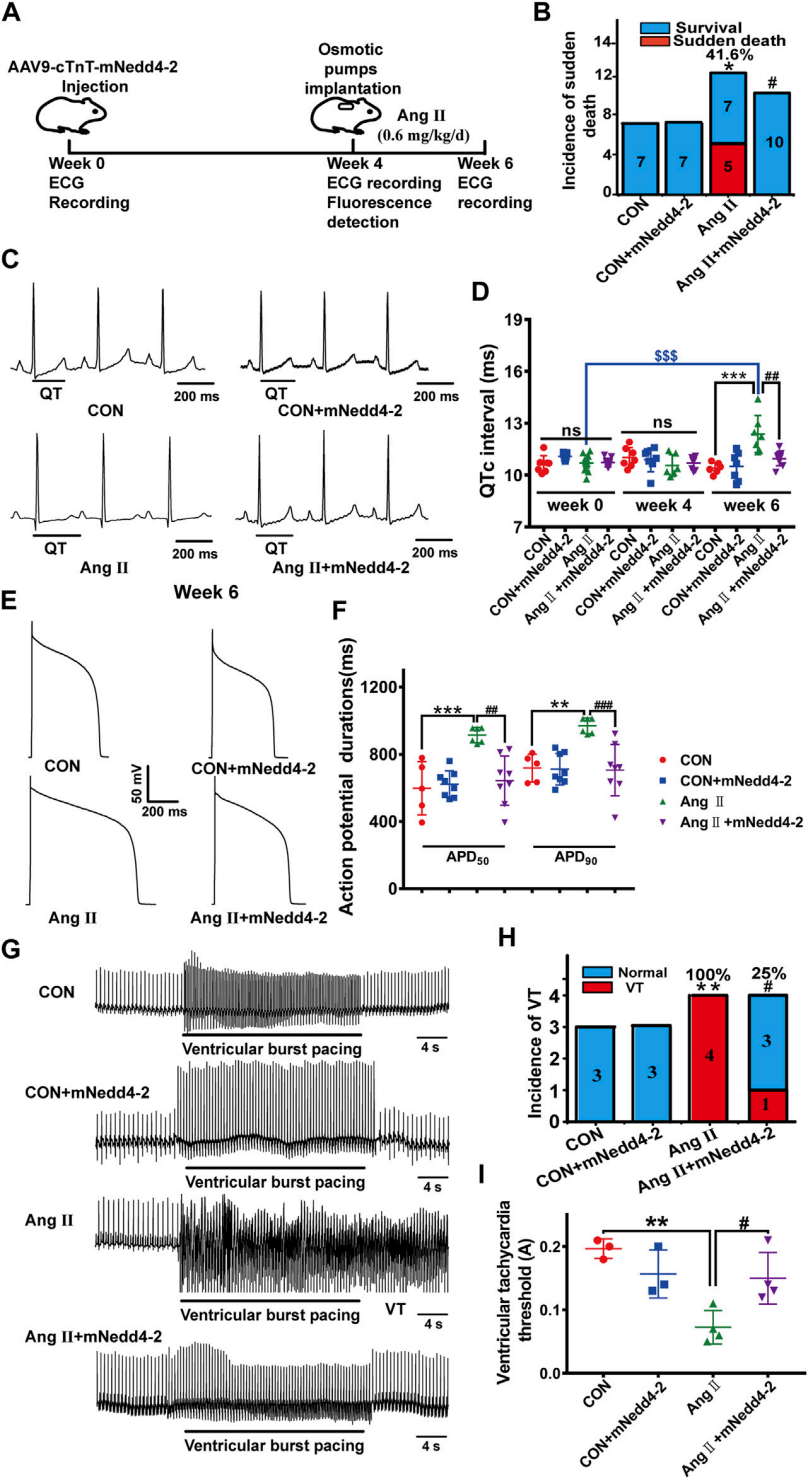
Overexpression of inactive Nedd4-2 (mNedd4-2) in guinea pigs prevented Ang II-induced electrical remodeling in *in vivo* and *ex vivo* heart. **(A)** Schematic diagram of mNedd4-2 treatment. **(B)** Summary data for the incidence of sudden death. **(C)** Representative *in vivo* ECG recordings at week 6 (end of the treatment) (*n* = 6–10 guinea pigs each group). QT interval measurements were labeled. **(D)** Summary data for the quantification of QTc intervals. **(E)** Representative action potential traces recorded from isolated ventricular myocytes. **(F)** Corresponding summary data for APD_50_ and APD_90_ (*n* = 5-8 from 3 hearts in each group). **(G)** Representative ECG tracings recorded from isolated perfused hearts under burst pacing. **(H)** Incidence of induced ventricular tachycardia with an intensity of 120 mA. **(I)** The ventricular tachycardia threshold (VTT) among different groups (*n* = 3–4). *p < 0.05, **p < 0.01, ***p < 0.001 *versus* CON; ^#^ *p* < 0.05, ^##^ *p* < 0.01, ^###^ *p* < 0.001 *versus* Ang II; ^$$$^ *p* < 0.001 *versus* week 0. ns: not statistically significant.

Based on the findings mentioned above, we proposed that suppressing Nedd4-2 attenuates the downregulation of *I*_Kr_ (Kv11.1/hERG channel) and thus alleviates cardiac electrical remodeling in pCH. We tried different approaches in the following experiments to suppress Nedd4-2 activity in pCH model. Several reports have demonstrated that activation of SGK1 enhances the expression level of mature hERG channels by inhibiting Nedd4-2-dependent ubiquitination (Maier et al., 2006; Lamothe and Zhang, 2013). A study has shown that a short-chain sphingolipid, C4-ceramide (C4-CER) can directly activate SGK1 (Caohuy et al., 2014). Therefore, we first tested the effect of C4-CER on electrical remodeling in pCH. C4-CER at 1, 5, 10 mg/kg/day i.p. for 2 weeks in healthy guinea pigs had no effect on QTc intervals (Supplementary Figure S3A,B). Administration of C4-CER at a dose of 5 mg/kg/d i.p. for 2 weeks did not prevent Ang II-treated guinea pigs from the incidence of sudden death (Supplementary Figure S3C). Concomitantly, C4-CER treatment had no effect on the prolongation of QTc intervals (Supplementary Figure S3D,E). The result demonstrated that SGK1 activator failed to prevent electrical remodeling in hypertrophied hearts of guinea pigs. We then directly suppressed Nedd4-2 by overexpressing the inactive catalytic form of Nedd4-2, Nedd4-2 C801S mutant (mNedd4-2) in the guinea pig hearts (Jespersen et al., 2007). An AAV9 vector containing mNedd4-2 C801S and eGFP sequences driven by cardiac-specific cTnT promoter was constructed and injected into guinea pig’s paw vein (Figure 3A). Four weeks after injection, recombinant vector was exclusively detected in the heart by fluorescence detection (Supplementary Figure S4). Ang II was then administrated for 2 weeks to induce pCH. Compared with pCH guinea pigs with an incidence of sudden death of 41.6% (5 of 12), all guinea pigs subjected to overexpression of mNedd4-2 alone or mNedd4-2 plus Ang II treatment were survived (Figure 3B). Furthermore, overexpression of mNedd4-2 did not affect QTc intervals in control guinea pigs, manifested by similar QTc intervals at 4 and 6 weeks (Figures 3C,D). Application of Ang II for 2 weeks induced a remarkable prolongation in QTc intervals, whereas overexpression of mNedd4-2 in pCH guinea pigs did not display QTc prolongation (Figures 3C,D). Overexpression of mNedd4-2 did not change APD_50_ and APD_90_ values of action potentials recorded from isolated ventricular myocytes in control guinea pigs, but it abolished the APD prolongation observed in the pCH guinea pigs (Figures 3E,F). Furthermore, a 120-mA stimulation did not induce ventricular tachycardia in isolated Langendorff-perfused hearts (Figure 3G) from control or mNedd4-2 expression guinea pigs, but it triggered ventricular tachycardia arrhythmias in all of 4 hearts from pCH guinea pigs (100%). Overexpression of mNedd4-2 reduced the occurrence of pacing-induced arrhythmias to 1 of 4 hearts (25%) in pCH guinea pigs (Figure 3H). In addition, overexpression of mNedd4-2 significantly elevated the VTT in hearts from pCH guinea pigs (Figure 3I). These findings indicated that cardiac overexpression of mNedd4-2 significantly reduced the susceptibility to arrhythmias in pCH.

**FIGURE 4:**
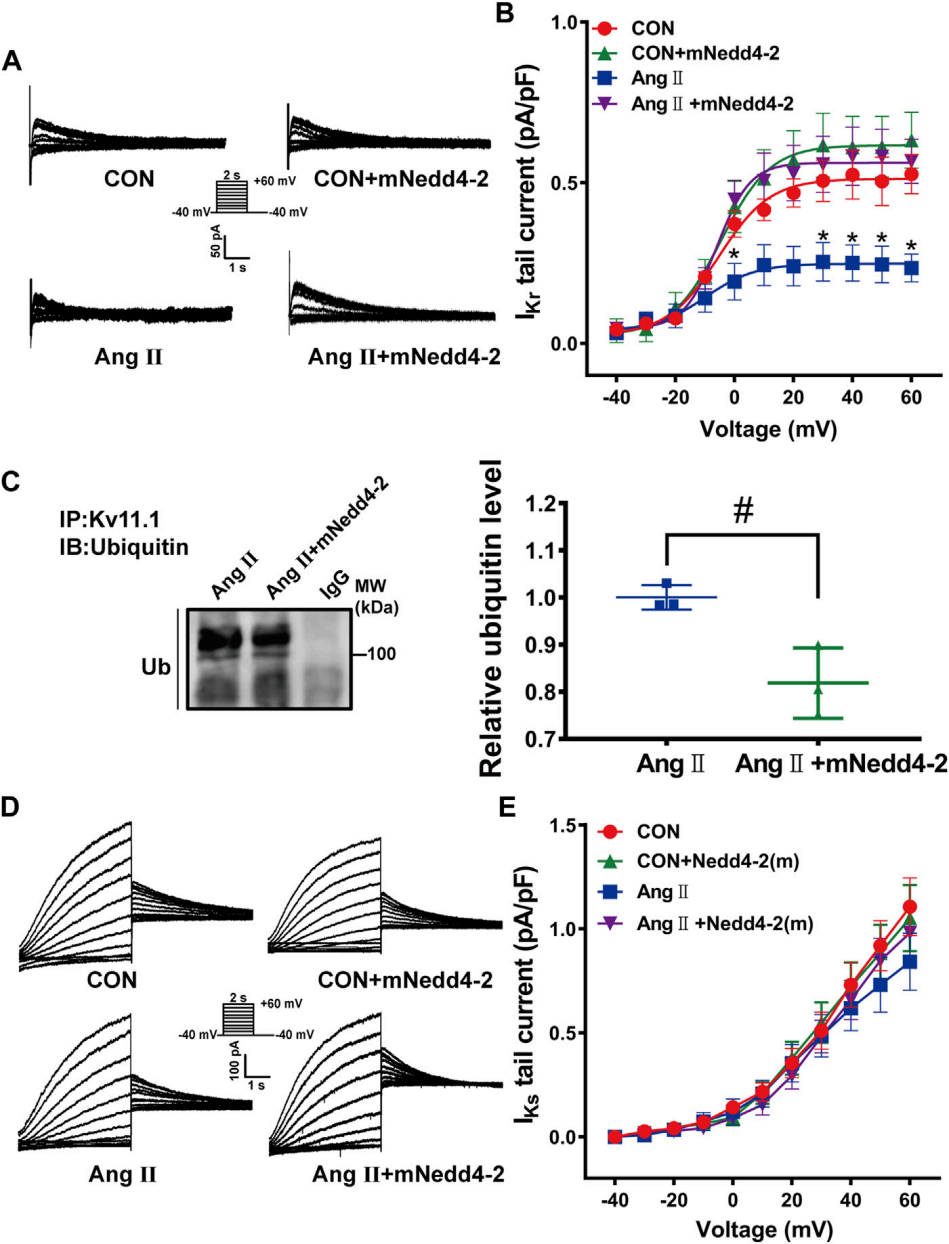
Overexpression of mNedd4-2 prevented the downregulation of Kv11.1 channels. **(A)** Representative *I*_Kr_ tail current tracings elicited by voltage pulses shown in the inset from isolated ventricular myocytes. **(B)** Quantification of *I*_Kr_ currents density-voltage relationship (*n* = 9–24 from 3 to 5 hearts in each group). **(C)** Representative immunoblots showed that left ventricular tissue proteins were pulled down by anti-Kv11.1 or anti-IgG antibodies and probed by anti-ubiquitin antibody (*n* = 3). **(D)** Representative *I*_Ks_ currents elicited by a pulse protocol shown in the inset. **(E)** Summary data of *I*_Ks_ currents density-voltage relationship (*n* = 14–30 from 3 to 5 hearts in each group). *p < 0.05 *versus* CON, ^#^ *p* < 0.05 *versus* Ang II.

Then, we further determined the effect of overexpression of mNedd4-2 on *I*_Kr_ currents in isolated cardiomyocytes. Representative *I*_Kr_ tail current traces from different groups were shown in Figure 4A. Overexpression of mNedd4-2 did not change *I*_Kr_ current densities in cardiomyocytes in control guinea pigs, but it significantly recovered the reduced *I*_Kr_ currents in pCH guinea pigs (Figure 4B). Co-immunoprecipitation analysis demonstrated that overexpression of mNedd4-2 significantly decreased the ubiquitination level of Kv11.1 protein (Figure 4C). Both Kv11.1 and Kv7.1 contain Nedd4-2 binding PY motif. However, overexpression of mNedd4-2 did not affect Kv7.1/*I*_Ks_ currents recorded from control and pCH guinea pigs (Figures 4D,E). These data indicated that overexpression of inactivated form of Nedd4-2 prevented the *I*_Kr_ reduction probably through inhibiting ubiquitination of Kv11.1 protein in pCH guinea pig myocardium. Moreover, echocardiography showed that mNedd4-2 did not affect ventricular wall thickness in control guinea pigs, whereas it reduced the systolic wall thickness of hypertrophic hearts but not the diastolic wall thickness (Supplementary Figure S5). The result indicated that inhibition of Nedd4-2 activity partially mitigated hypertrophic remodeling.

### Effect of pharmacologically suppressing Nedd4-2 on electrical remodeling

**FIGURE 5:**
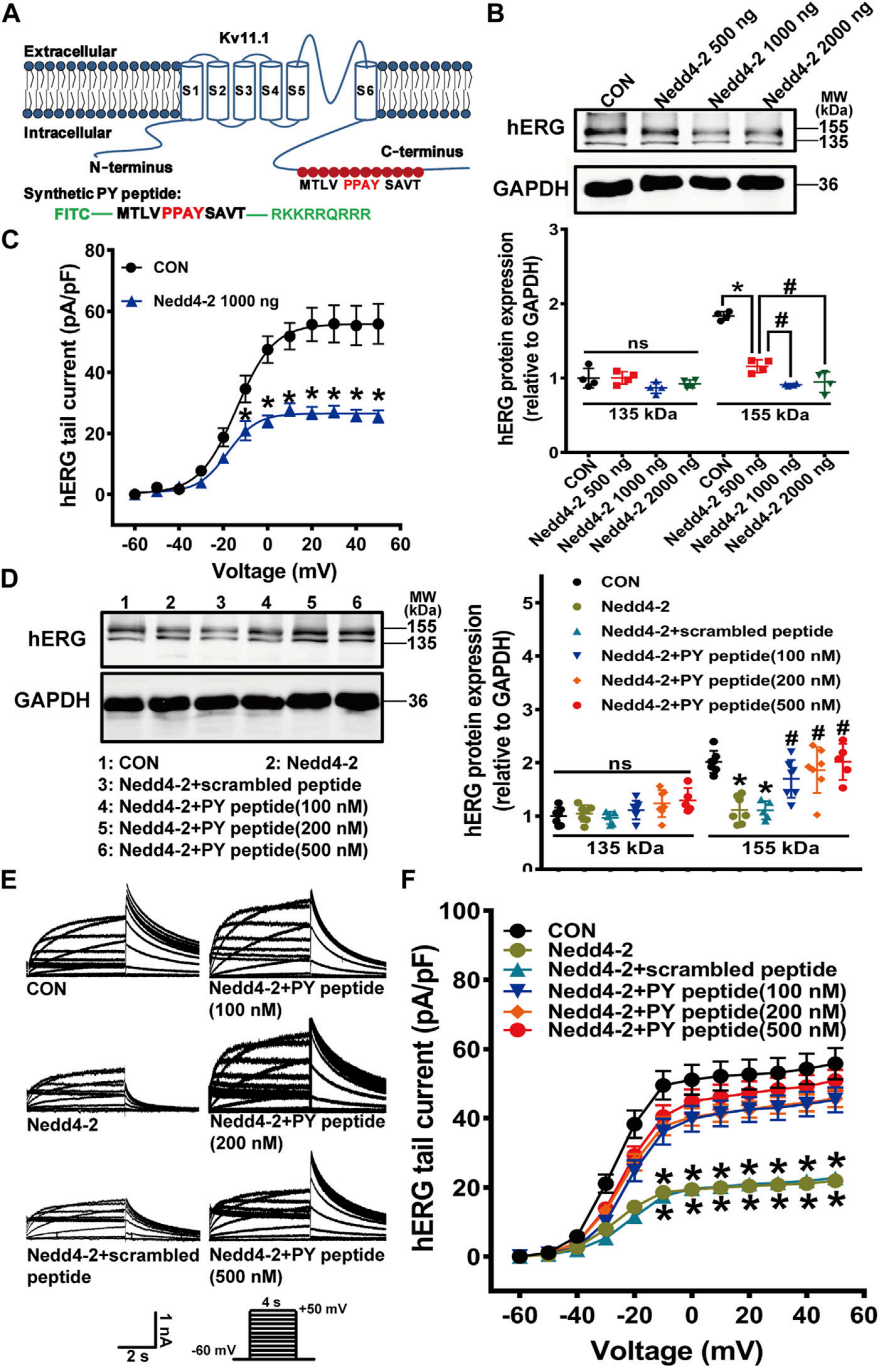
The synthetic PY peptide prevented Nedd4-2-dependent degradation of the hERG channel expressed in hERG-HEK293 cells. **(A)** Diagram showing that the amino acid sequence of Nedd4-2 targeting PPAY motif is located in C-terminal region of hERG channel, and a synthetic polypeptide chain with the same sequence of 12 amino acids containing PPAY. **(B)** Representative immunoblots of hERG protein and quantification of the band intensities showing the effect of Nedd4-2 overexpression on the expression of hERG protein (*n* = 4, *p < 0.05, ^#^ *p* < 0.05). **(C)** Summary data for tail current density-voltage relationship (*n* = 16–26 cells in each group). **(D)** Representative immunoblots (left panel) and quantification of band density (right panel) (*n* = 5–7 samples in each group). **(E)** Representative hERG current tracings elicited by the voltage protocol in the inset. **(F)** Corresponding summary data of hERG current density- voltage relationships (*n* = 15–24 cells in each group). *p < 0.05 *versus* CON; ^#^ *p* < 0.05 *versus* Nedd4-2 alone. ns: not statistically significant.

Because Nedd4-2 has many downstream targets and suppression of Nedd4-2 activity might affect off-target proteins, we further designed a short-length peptide, composed of identical amino acid PY sequence in the C-terminus of Kv11.1 protein and fused this peptide with a cell-penetrating peptide. We supposed this synthetic PY peptide competitively binds to Nedd4-2 protein to selectively interfere the Nedd4-2-dependent ubiquitination of membrane Kv11.1 channel (Figure 5A). We first tested if this synthetic PY peptide attenuated Nedd4-2-dependent degradation of the hERG channel in the HEK293 cell line with stable transfection of the hERG channel (hERG-HEK293). Transfection of Nedd4-2 plasmids at 500, 1,000, and 2,000 ng into hERG-HEK293 cells significantly reduced the expression of mature hERG protein (155 kDa) and decreased hERG currents (Figures 5B,C). A maximal reduction of hERG expression and hERG currents was observed when Nedd4-2 plasmid was transfected at a dose of 1,000 ng. Thus, this dose was used in the subsequent experiments. The synthetic PY peptide was delivered into the hERG-HEK293 cells after 12 h incubation, as indicated by the presence of the TRITC marker, which was conjugated to the synthetic PY peptide (Supplementary Figure S6). The synthetic PY peptide at the concentrations tested significantly alleviated Nedd4-2-induced reduction of hERG proteins and currents in hERG-HEK293 cells (Figures 5D–F). The scrambled peptide did not affect Nedd4-2-induced reduction of hERG proteins and currents in hERG-HEK293 cells.

**FIGURE 6:**
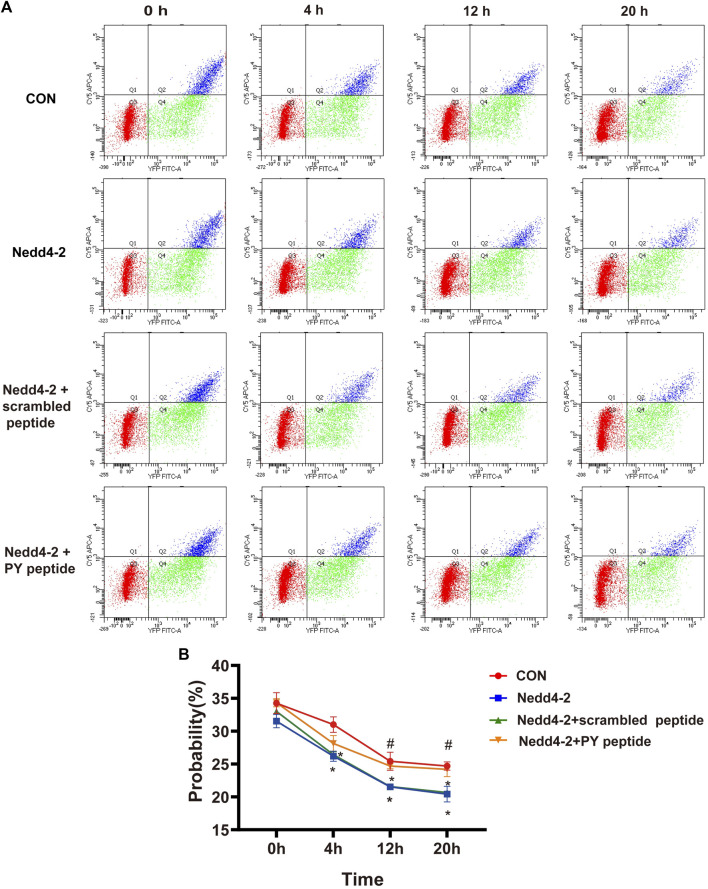
Flow cytometry analysis of membrane hERG channels. **(A)** Based on the analyses of single-color controls, vertical and horizontal lines divided YFP- and BTX-CY5-positive cells, respectively. Representatives are YFP-positive cells with BTX-CY5 signal above (blue dots) or below threshold (green dots) and untransfected cells (red dots). **(B)** Time course of BBS-hERG-YFP delivery to the surface. The curves show probability in different times of 0, 4, 12 and 20 h treatment on BBS-hERG-YFP-HEK293 cells with BFA (*n* = 3). *p < 0.05 *versus* CON, ^#^ *p* < 0.05 *versus* Nedd4-2.

To determine the mechanism of action of the synthetic PY peptide, we further detected the change of surface hERG channels in the presence of the PY peptide using flow cytometry analysis. HEK cells transiently transfected with WT-BBS-hERG-YFP displayed robust signals for total (YFP) and surface (BTX-CY5) fluorescence (Figure 6). After WT-BBS-hERG-YFP was transfected for 24 h, Nedd4-2 plasmid and synthetic PY peptide were applied. Brefeldin A (BFA) was added to inhibit the transportation of newly synthetic proteins to the cell membrane. At 4, 12 and 20 h, the proportion of both YFP- and CY5-positive cells (representing mature hERG channel) in the Nedd4-2 overexpression group were significantly lower than that in the control group (Figure 6). These data indicated that overexpression of Nedd4-2 significantly increased the degradation of mature hERG protein. The synthetic PY peptide, but not scrambled PY peptide, significantly increased the proportion of both YFP and CY5 positive cells at 12 and 20 h (Figure 6B). Our finding provided direct evidence that the synthetic PY peptide prevents hERG channel proteins from Nedd4-2-dependent degradation.

**FIGURE 7:**
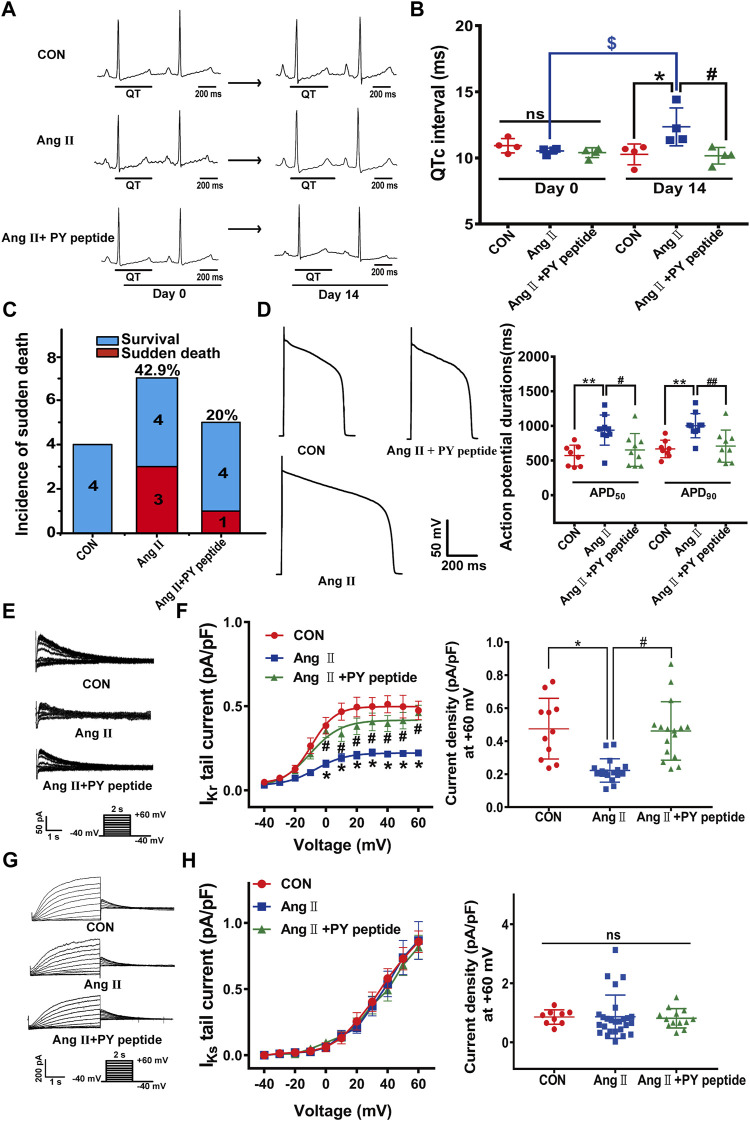
The synthetic PY peptide prevented cardiac electrical remodeling in pCH guinea pigs. **(A)** Representative *in vivo* ECG recordings. QT interval measurements were labeled. **(B)** Summary data for the quantification of QTc intervals (*n* = 4 in each group). **(C)** Incidence of sudden death of animals. **(D)** Representative raw tracings of action potentials recorded in isolated ventricular cardiacmyocytes (left panel) and corresponding summary data for APD_50_ and APD_90_ (*n* = 8–10 myocytes from 4 hearts in each group). **(E)** Representative *I*_Kr_ tail current traces recorded under a pulse protocol in inset. **(F)** Corresponding summary data for *I*_Kr_ tail current density-voltage relationship followed by *I*_Kr_ tail current densities at +60 mV (*n* = 11–17 myocytes recorded from 4 hearts in each group). **(G)** Representative *I*_Ks_ current traces recorded under pulse protocol shown in inset. **(H)** Summary data of *I*_Ks_ tail currents density-voltage relationship followed by *I*_Ks_ tail current density at +60 mV (*n* = 10–24 cardiomyocytes recorded from 4 hearts in each group). *p < 0.05, **p < 0.01 *versus* CON; ^#^ *p* < 0.05, ^##^ *p* < 0.01 *versus* Ang Ⅱ; ^$^ *p* < 0.05 *versus* day 0. ns: not statistically significant.

Next, we tested the effect of *in vivo* administration of synthetic PY peptide on cardiac electrical remodeling in pCH guinea pigs. Intravenous injection of the synthetic PY peptide (0.23 mg/kg every 2 days for 14 days) shortened the prolonged QTc intervals in pCH to a level as shown in control guinea pigs (Figures 7A,B). In addition, the synthetic PY peptide decreased the incidence of sudden death from 42.9% (3 of 7) to 20% (1 of 5) (Figure 7C). The action potential recordings revealed that synthetic PY peptide significantly restored the prolonged APD_50_ and APD_90_ in isolated cardiomyocytes from pCH guinea pigs (Figure 7D). Furthermore, patch-clamp recordings showed that the synthetic PY peptide significantly mitigated the *I*_Kr_ reduction in pCH cardiomyocytes (Figures 7E,F). However, the synthetic PY peptide treatment did not affect *I*_Ks_ currents in control and pCH guinea pigs (Figures 7G,H). Meanwhile, synthetic PY peptide treatment alone did not change QTc intervals nor cause sudden death (Supplementary Figure S7). These data suggest that the synthetic PY peptide effectively prevents the electrical remodeling in pCH.

## Discussion

The significant finding of the study is that Nedd4-2-dependent ubiquitination is critically involved in the downregulation of Kv11.1/*I*_Kr_ in pCH. More importantly, our newly developed synthetic PY peptide can prevent cardiac electrical remodeling and decrease the occurrence of cardiac arrhythmias in pCH by suppressing Nedd4-2-dependent ubiquitination.

In this study, significant prolongations of APD and QTc intervals in pCH were associated with a high susceptibility to ventricular arrhythmias and sudden death. The reduction of *I*_Kr_, but not *I*_Ks_, was identified in ventricular cardiomyocytes of pCH guinea pigs and thus constituted the molecular basis of repolarization delay. A previous study has shown that *I*_Ks_, but not *I*_Kr_, recorded from ventricular cardiomyocytes is reduced in Ang II-induced pCH (Zankov et al., 2019). The ejection fraction was significantly decreased in that study but remained unchanged in the current study in pCH guinea pigs. Thus, the diversity of ion channel alterations is likely due to different stages of pathological conditions.

We found that the degradation mediated by Nedd4-2-dependent ubiquitination of Kv11.1 protein was enhanced in hypertrophic myocardium. In addition, we provided data showing that this Nedd4-2-dependent ubiquitination of Kv11.1 protein primarily contributed to a reduction of *I*_Kr_ density and subsequently was involved in cardiac electrical remodeling. This notion was strongly supported by our findings that the transcription level of the *KCNH2* gene encoding Kv11.1 was not altered, but the abundance of Kv11.1 protein was decreased in hypertrophic myocardium. Moreover, we found that increased Nedd4-2 activity and its interaction with Kv11.1 led to a reduction of mature Kv11.1 protein abundance. The finding is consistent with a previous report that Nedd4-2 expression is significantly increased in rat heart failure model and *in vitro* hypertrophied neonatal rat cardiomyocytes (Luo et al., 2017). The previous study has demonstrated that overexpression of Nedd4-2 selectively targets the mature hERG channel proteins in heterologous expression cells, although both immature and mature channels contain the PY motif (Cui and Zhang, 2013). Our previous study has also shown that Ang II significantly decreased mature hERG channel protein levels without affecting the immature hERG channel protein level *via* accelerating degradation of the channel on the surface without affecting the immature hERG channel protein level (Cai et al., 2014). However, in the current study both immature and mature Kv11.1 channels were downregulated in pCH. The reduction of immature Kv11.1 channel implies an enhanced degradation of the immature channel protein, which probably occurs at endoplasmic reticulum (ER) level as previous study has reported (Lamothe and Zhang, 2016). In addition to the Kv11.1 channel, Kv7.1 is also the substrates of Nedd4-2-dependent ubiquitination as it contains a conserved PY motif. Previous studies have demonstrated that Nedd4-2-dependent ubiquitination physiologically regulated the surface density of Kv7.1 (KCNQ1) channels (Delisle et al., 2009; Andersen et al., 2015). However, we did not observe an alteration of interaction between Nedd4-2 and Kv7.1 channels. Instead, an increase of KCNQ1 mRNA expression was observed and this may be a secondary response to the delayed repolarization induced by *I*_Kr_ reduction. The result hinted that the high expression of Nedd4-2 may selectively bind its substrate under pathological conditions. The underlying mechanism remains unclear and is warranted to be determined in future studies.

Based on the findings mentioned above that Nedd4-2-dependent ubiquitination of Kv11.1 protein is critically involved in cardiac electrical remodeling in pCH guinea pigs, we developed different approaches to intervene the remodeling by suppressing Nedd4-2 activity effectively. We found that SGK1 activator had no protectiveness on the prolonged QTc intervals and sudden death in pCH guinea pigs although previous studies have demonstrated that SGK1 activation increases hERG protein expression in HEK cell by inhibiting Nedd4-2 activity as well as promoting Rab11-mediated hERG recycling (Maier et al., 2006; Lamothe and Zhang, 2013). The observation indicated that the findings about SGK1 activator under physiological conditions were not translational. In addition, SGK1 itself may induce cardiac hypertrophy and promote arrhythmia (Das et al., 2012; Bezzerides et al., 2017). We then used an alternative approach to directly suppress Nedd4-2 activity through myocardial-specific overexpression of mutant catalytically inactive form of Nedd4-2, Nedd4-2 (C801S) (Scheffner et al., 1995). The result showed that the approach significantly reduced Nedd4-2-dependent ubiquitination of Kv11.1, increased *I*_Kr_ currents, and then prevented electrical remodeling in pCH guinea pigs. It should be noted that suppressing Nedd4-2 activity by overexpression of mutant catalytically inactive form of Nedd4-2 did not change *I*_Kr_ current density in normal guinea pigs, suggesting that the Nedd4-2 activity is minimal in physiological conditions. These findings suggest that Nedd4-2 may be a target to treat arrhythmias in pCH effectively.

Given that Nedd4-2 plays many roles in maintaining proteostasis, inhibition of Nedd4-2 may lead to some confounding off-target effects. Thus, we further designed a peptide containing the same PY sequence located on the C-terminals of Kv11.1 protein that binds to Nedd4-2 protein. We found that in cultured HEK cells with stable expression of hERG channels, the synthetic PY peptide profoundly alleviated Nedd4-2-induced reduction of functional hERG channels. Flow cytometry analysis provided further evidence showing that the PY peptide significantly prevented hERG channel proteins from degradation induced by overexpression of Nedd4-2. Our finding suggests that this PY peptide competitively occupies the binding sites of Nedd4-2 required for Kv11.1 ubiquitination. In addition, *in vivo* administration of this synthetic PY peptide significantly increased *I*_Kr_ currents and subsequently shortened the prolongation of QTc intervals and APD, decreasing the incidence of sudden death in pCH without affecting ECG parameters in control animals. Taken together, our findings suggest that this synthetic PY peptide is a promising pharmacological agent to prevent the Nedd4-2-dependent deficiency of Kv11.1 channel in cardiac hypertrophy.

Because Kv11.1/hERG channel is impaired in inherited or drug-induced acquired long QT syndrome, many hERG activators have been developed and some of those effectively exert antiarrhythmic action (Su et al., 2021). However, the potential proarrhythmic risk of hERG channel activators still exists (Su et al., 2021). Our synthetic PY peptide competitively inhibits the degradation of the Kv11.1 channel, which is over-activated because of higher expression of Nedd4-2 under pathological conditions. Therefore, we believe it is a novel pharmacological approach specifically targeting Nedd4-2-dependent ubiquitination of hERG channels in pCH. Intriguingly, a similar strategy has been recently reported for stabilizing Kv7.1 channel proteins on cell membrane through engineered deubiquitinases (Kanner et al., 2020).

We conclude that this study reveals that increases in Nedd4-2 activity and Nedd4-2-dependent ubiquitination are critical causal factors for downregulating the Kv11.1 channels in pCH. Based on our findings, we have successfully developed a synthetic PY peptide to rescue the reduced Kv11.1 channels and *I*_Kr_ currents and the subsequential cardiac electrical remodeling. This intervention represents a novel strategy to prevent *I*_Kr_-deficient relevant arrhythmia and sudden death under pathological condition.

## Data Availability

The original contributions presented in the study are included in the article/Supplementary Material, further inquiries can be directed to the corresponding authors.

## Abbreviations

Ang II, angiotensin II; APD, action potential duration; cTnT, cardiac troponin T; hERG, human ether-á-go-go-related gene; pCH, pathological cardiac hypertrophy; SCD, sudden cardiac death; PES, programmed electrical stimulation; SGK, serum- and glucocorticoid-inducible kinases; Ub, Ubiquitin; VT, ventricular tachycardia.
