# Supplementary material for: Pharmacological suppression of Nedd4-2 rescues the reduction of Kv11.1 channels in pathological cardiac hypertrophy
Source: Front Pharmacol. 2022 Aug 17;13:942769. doi: 10.3389/fphar.2022.942769 (PMC9428276; doi:10.3389/fphar.2022.942769)
Supplement: Supplementary file 1 [file DataSheet1.docx]

**Pharmacological suppression of Nedd4-2 rescues the reduction of Kv11.1 channels in pathological cardiac hypertrophy**

Hua Zhang^1†^, Tian Fu^1†^, Jinglei Sun^1^, Sihao Zou^1^, Suhua Qiu^1^ Jiali Zhang^1^, Shi Su^1^, Chenxia Shi^1^, De-Pei Li^2*^, Yanfang Xu^1*^

^1^Department of Pharmacology, Hebei Medical University; The Key Laboratory of New Drug Pharmacology and Toxicology, Hebei Province. Shijiazhuang 050017, China

^2^Center for Precision Medicine, Department of Medicine, School of Medicine University of Missouri, Columbia MO 65212, USA

^†^These authors have contributed equally to this work.

**Running title: Intervention of K^+^ channel downregulation**

*Correspondence:

Yanfang Xu

yanfangxu@hebmu.edu.cn

De-pei Li

lidep@health.missouri.edu

Supplementary Materials

**Supplementary Table 1: Echocardiography parameters in control (CON) and Ang II-treated hearts.**

| Parameters | CON (n=5) | Ang II (n=7) |
| --- | --- | --- |
| LVAW; d | 1.34±0.057 | 1.7±0.064** |
| LVAW; s | 1.67±0.024 | 2.07±0.055*** |
| LVPW; d | 1.21±0.051 | 1.6±0.058*** |
| LVPW; s | 1.63±0.025 | 2.01±0.06*** |
| LVID; d | 5.55±0.47 | 6.09±0.63 |
| LVID; s | 3.27±0.62 | 4±0.51 |
| IVS; d | 0.75±0.072 | 0.98±0.027** |
| IVS; s | 0.93±0.066 | 1.18±0.028** |
| EF (%) | 71.16±7.73 | 63.66±4.01 |
| FS (%) | 42.52±5.97 | 35.7±2.97 |

d: diastolic; s: systolic; LVAW: Left ventricular anterior wall (mm); LVPW: Left ventricular posterior wall (mm); IVS: Interventricular septum (mm); LVID: Left ventricular inner diameter (mm); EF: ejection fraction; FS: short axis fractional shortening ***P*<0.01, ****P*<0.001 compared with values in CON.

**Supplementary Figures S1-S7**


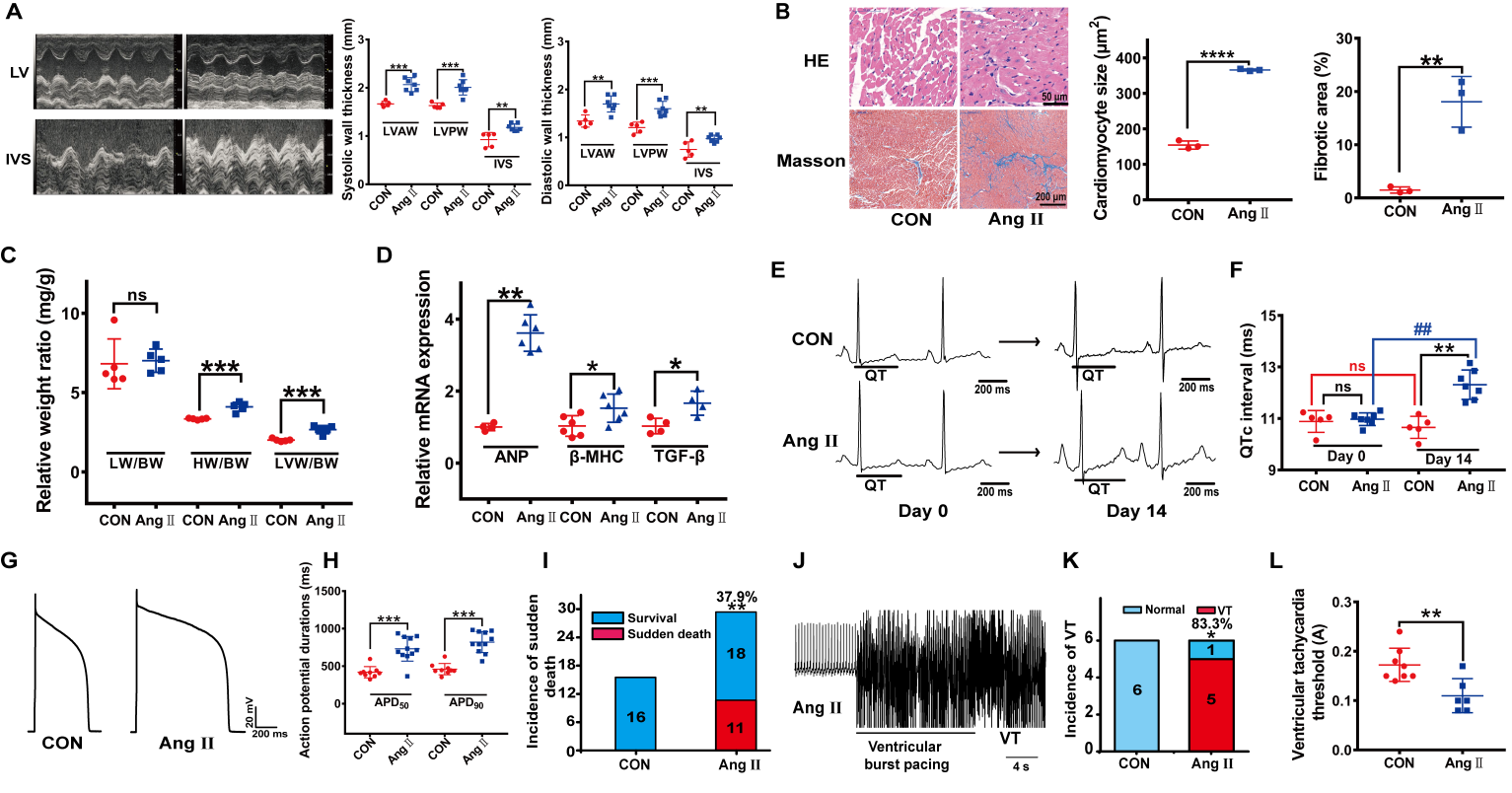


**Figure S1.** **Ang II-induced pathological cardiac hypertrophy (pCH) and electrical remodeling in guinea pigs.** A: Representative M-type echocardiography and summary data for systolic and diastolic wall thickness. LVAW: the left ventricular anterior wall; LVPW: left ventricular posterior wall; IVS: interventricular septum (CON n=5, Ang II n=7). B: Representative HE and Masson staining of myocardial tissues and corresponding summary data for myocyte size and myocardial fibrosis area (n=3). C: Relative weight ratio of heart, lung, and ventricle. LW: lung weight, HW: heart weight, BW: body weight, LV: left ventricular (n=5). D: Relative mRNA expression of hypertrophic related factors, presented as fold changes compared with the control (CON). The values were normalized to an internal standard GAPDH (n=4). E: Representative *in vivo* electrocardiograph (ECG) recorded from guinea pigs at day 0 and day 14. F: Summary data for heart rate-corrected QT intervals. G: Representative action potential tracings recorded in ventricular cardiomyocytes. H: Summary data for action potential durations (CON: n=9 cells from 3 hearts, Ang II: n=11 cells from 5 hearts). I: Incidence of sudden death in control and Ang II-treated guinea pigs. J: Representative *ex vivo* ECG traces recorded from isolated perfused hearts under programmed electrical stimulation. K: Incidence of ventricular tachycardia under a 130 mA pacing. L: The ventricular tachycardia thresholds measured under programmed electrical stimulation (n=6-8). **P* < 0.05, ***P* < 0.01, ****P* < 0.001 versus CON; ^##^*P* < 0.01 versus day 0. ns: not statistically significant.


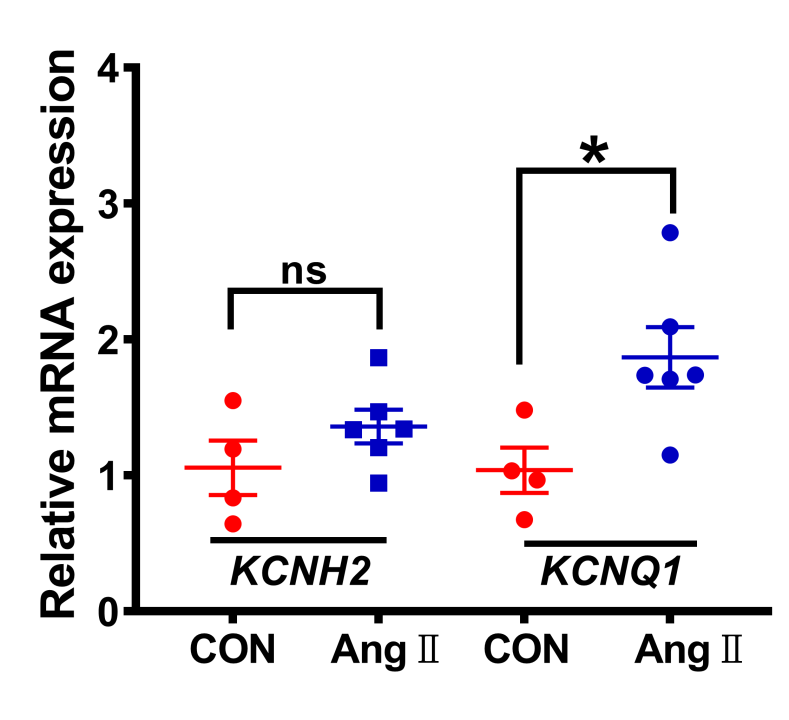


**Figure S2.** **Expression levels of *KCNH2* and *KCNQ1* mRNA in the myocardium.** Relative mRNA expression of *KCNH2* and *KCNQ1* were presented as fold changes of the mean value in the control group, and quantification was normalized by internal standard *GAPDH*. CON, n=4, Ang II, n=6. **P*<0.05 versus CON. ns: not statistically significant.


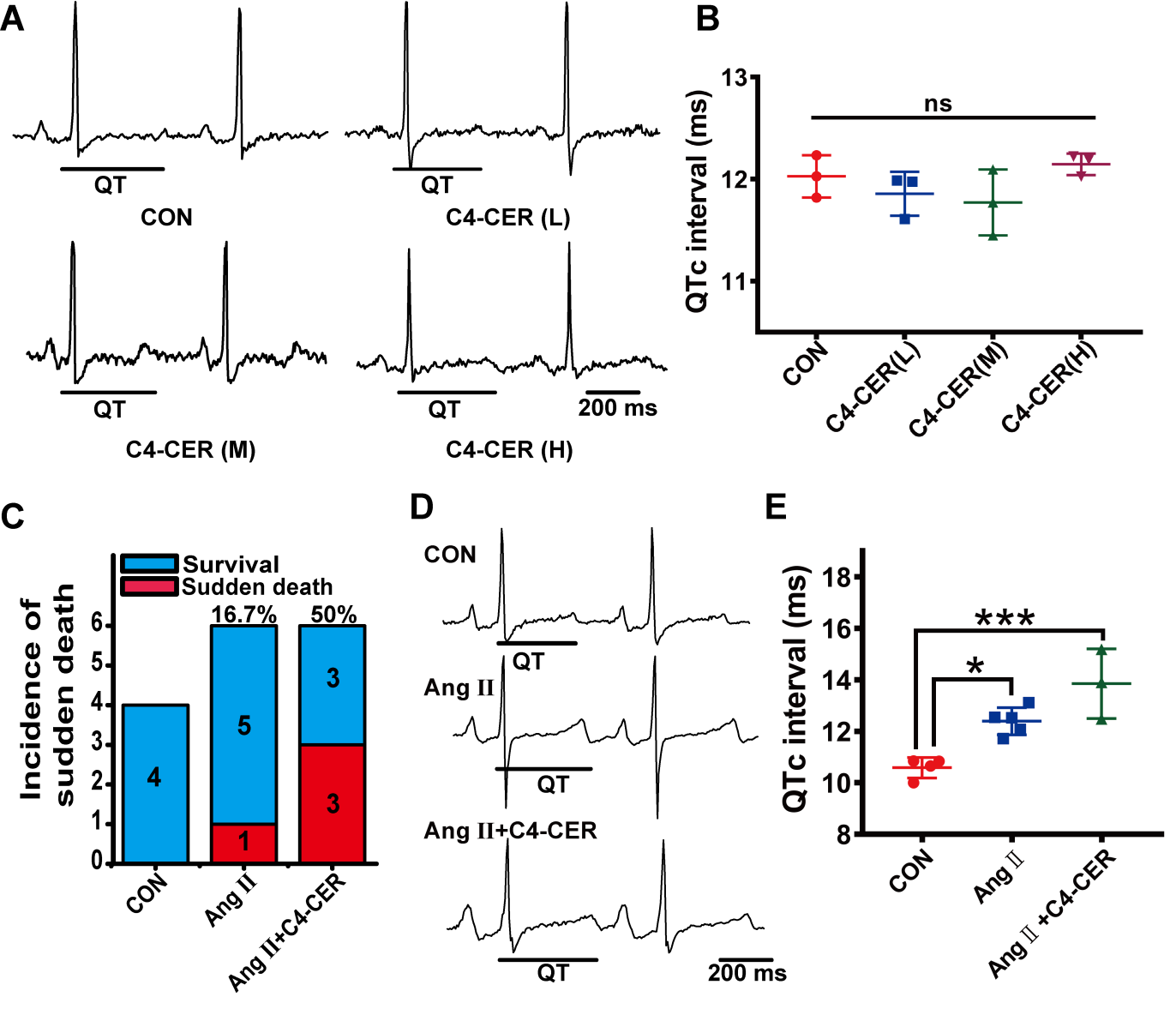


**Figure S3. Effects of SGK1 activation on cardiac electrical remodeling in guinea pigs.** A: Representative surface ECG recordings. B: Summary data for calculated heart rate-corrected QT intervals (QTc) in control and guinea pig treated by C4-CER at low dose (1 mg/kg/d), medium dose (5 mg/kg/d), and high dose (10 mg/kg/d). C: The incidence of sudden death in control (CON), pCH (Ang II), and C4-CER treated (5 mg/kg/d) pCH (Ang II +C4-CER) guinea pigs. D and E: Representative surface ECG recordings (D) and QTc intervals (E) calculation from 3 groups. **P* < 0.05, ****P* < 0.001versus CON. ns: not statistically significant.


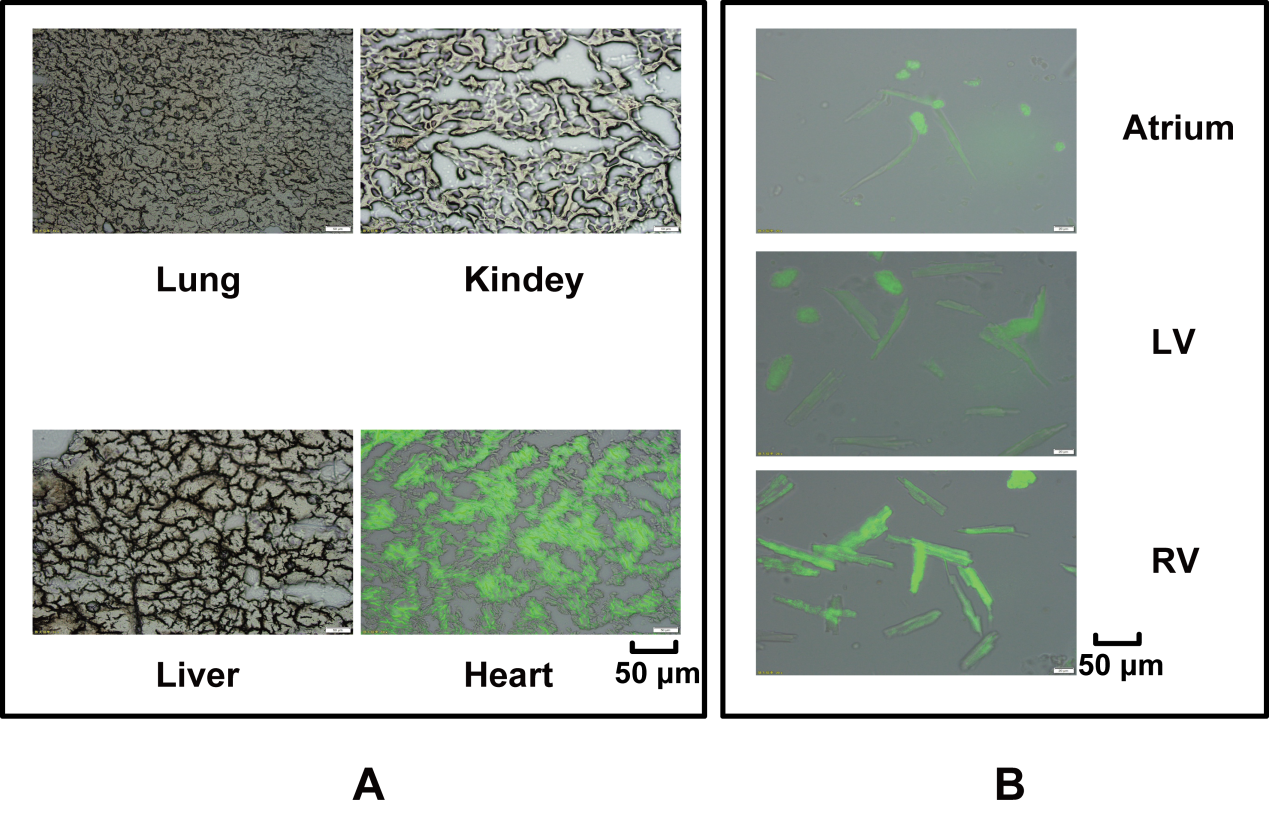


**Figure S4. The fluorescence expressions after administration of a recombinant AAV9-cTnT-mNedd4-2 virus.** A: eGFP expressions in different tissues 4 weeks after intravenous injection of viral vector into guinea pigs. The tissue slices at a thickness of 9 μm were sectioned and then observed under a fluorescence microscope. B: Fluorescence expression in acutely isolated cardiomyocytes in different parts of the heart under a fluorescence microscope. RV: right ventricular, LV: left ventricular.
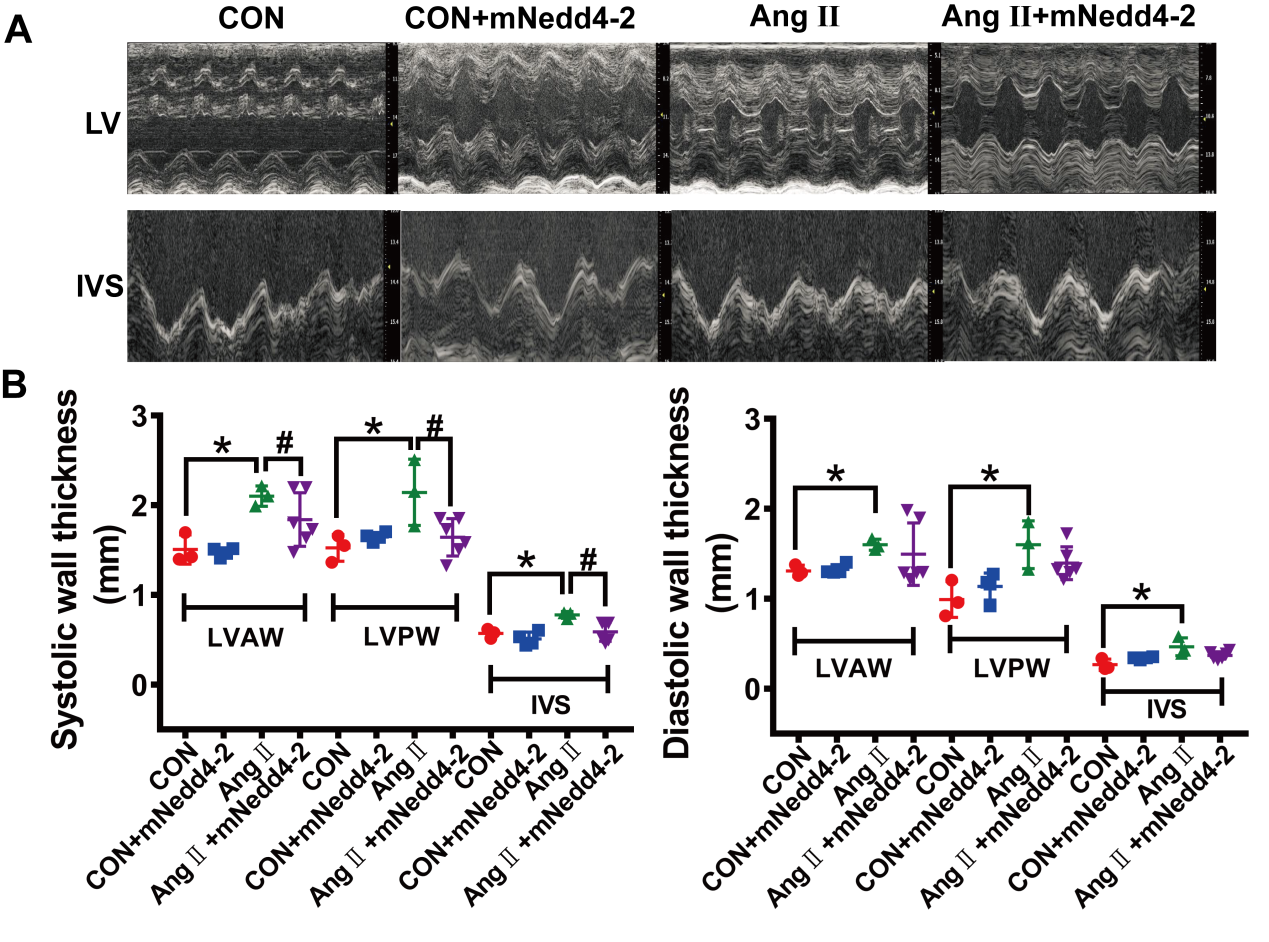


**Figure S5. Effects of overexpression of mNedd4-2 on ventricular wall thickness in guinea pigs.** A: Representative map of the M-type echocardiography. B: Summary data for wall thickness. LVAW: left ventricular anterior wall; LVPW: left ventricular posterior wall; IVS: interventricular septum. CON: control animals, n=3; CON+mNedd4-2: healthy guinea pigs treated with mNedd4-2, n=4. Ang II: hypertrophy model group, n=3; Ang II+mNedd4-2: Ang II combined with mNedd4-2, n=6. **P* < 0.05 versus CON group, ^#^*P* < 0.05 versus Ang II group.


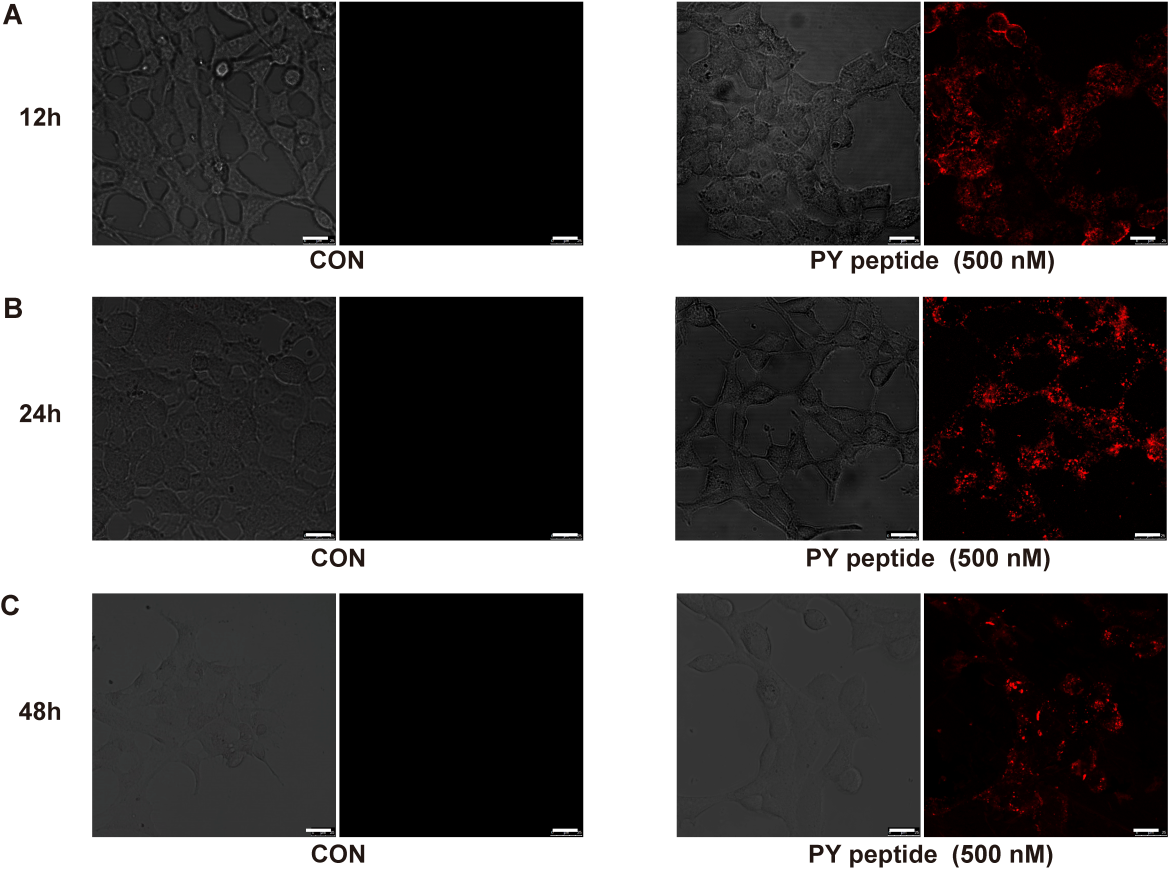


**Figure S6. Fluorescence images after hERG-HEK cells incubated with the synthetic PY peptides.** The synthetic PY peptide at the concentration of 500 nM was incubated in hERG-HEK cells for 12 h (A), 24h (B) and 48 h (C), respectively and then observed under a fluorescence microscope. Left panels: images under bright field; Right panels: images under florescent light.

**
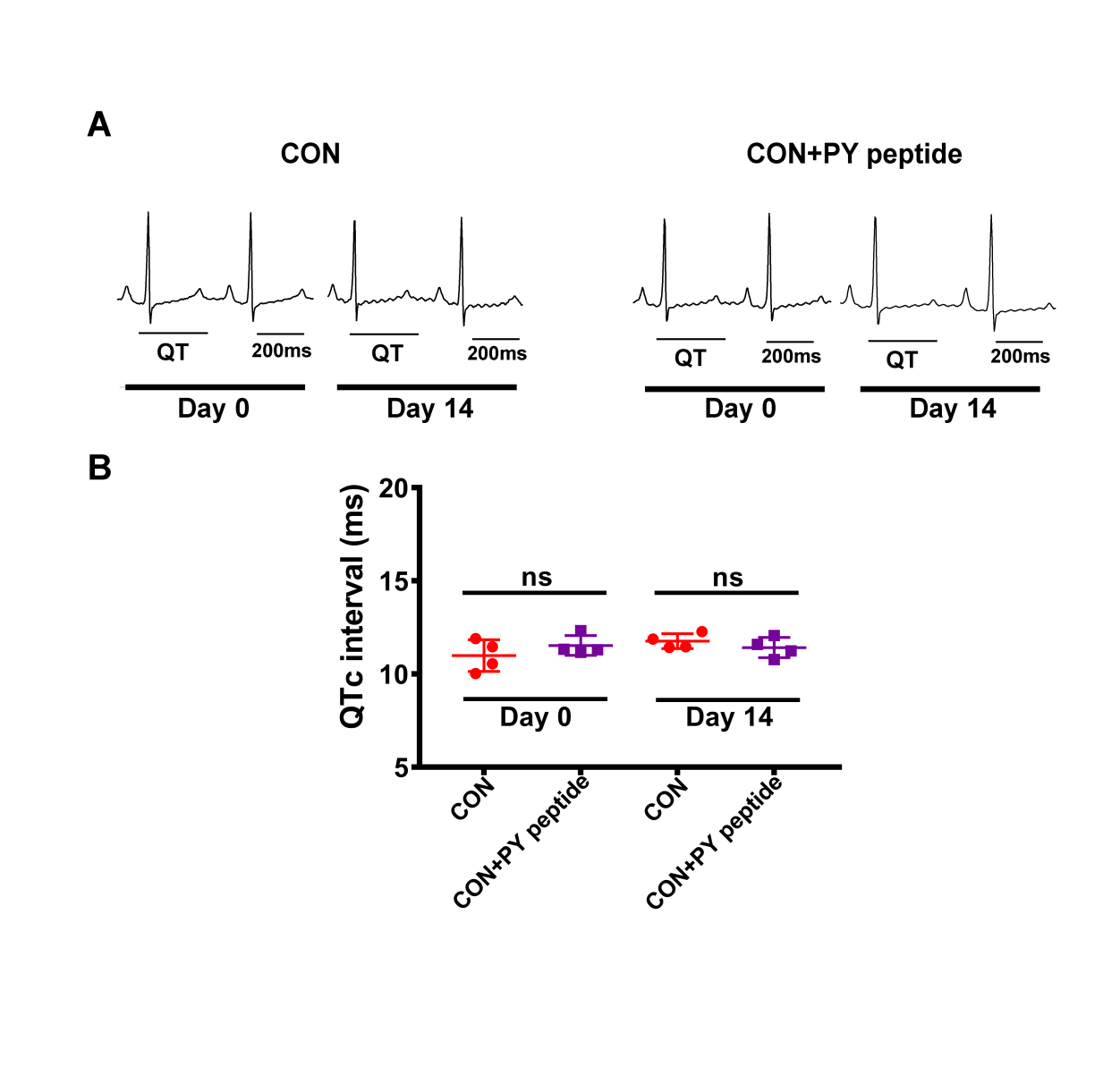
**

**Figure S7. Effects of synthetic PY peptide on ECG recordings in control healthy guinea pigs.** A: Representative lead II surface ECG traces recorded in anesthetized guinea pigs. B: Summary data for calculated QTc intervals. N=4 in each group. ns: not statistically significant.
